# Genome Sequences of Three Cluster AU *Arthrobacter* Phages, Caterpillar, Nightmare, and Teacup

**DOI:** 10.1128/genomeA.01121-17

**Published:** 2017-11-09

**Authors:** Tamarah L. Adair, Emily Stowe, Marie C. Pizzorno, Gregory Krukonis, Melinda Harrison, Baylor University SEA-PHAGES, Bucknell University SEA-PHAGES, Cabrini University SEA-PHAGES, Steven G. Cresawn, Rebecca A. Garlena, Daniel A. Russell, Welkin H. Pope, Deborah Jacobs-Sera, Graham F. Hatfull

**Affiliations:** a Department of Biology, Baylor University, Waco, Texas, USA; b Department of Biology, Bucknell University, Lewisburg, Pennsylvania, USA; c Department of Biological Sciences, Cabrini University, Radnor, Pennsylvania, USA; d Department of Biology, James Madison University, Harrisonburg, Virginia, USA; e Department of Biological Sciences, University of Pittsburgh, Pittsburgh, Pennsylvania, USA

## Abstract

Caterpillar, Nightmare, and Teacup are cluster AU siphoviral phages isolated from enriched soil on *Arthrobacter* sp. strain ATCC 21022. These genomes are 58 kbp long with an average G+C content of 50%. Sequence analysis predicts 86 to 92 protein-coding genes, including a large number of small proteins with predicted transmembrane domains.

## GENOME ANNOUNCEMENT

The *Actinobacteria* are a large and diverse group of soil bacteria with complex genetic relationships with each other and with their bacteriophages. An important genus of *Actinobacteria*, the *Arthrobacter*, includes common soil inhabitants that are important in biogeochemical cycling and bioremediation (1). Few *Arthrobacter* phages have been described relative to other *Actinobacteria* phages (2–4), and to better understand *Arthrobacter* phage diversity, students in the Science Education Alliance-Phage Hunters Advancing Genomic and Evolutionary Science (SEA-PHAGES) program used *Arthrobacter* sp. strain ATCC 20122 as a host to isolate and characterize bacteriophages from soil samples (5, 6). Here we report three newly discovered phages, Caterpillar, Nightmare, and Teacup, isolated on *Arthrobacter* sp. ATCC 20122, using enriched soil samples collected in Waco, TX, Chester, PA, and Lewisburg, PA, respectively. All three phages produce small clear plaques and have siphoviral virion morphologies with isometric heads 60 nm in diameter and flexible tails approximately 260 nm long.

Double-stranded DNA was extracted from high-titer phage lysates and sequenced on an Illumina MiSeq platform. Sequence reads from each genome were assembled into single contigs using Newbler and Consed (7), with minimum coverage of 160-fold. All genomes are members of cluster AU and have defined ends with 9-base complementary 3′ single-stranded DNA extensions (right end, 5′-CGCCGGCCT in Nightmare and Teacup and 5′-CGCCGGCCC in Caterpillar). The average G+C content for these three phages is 50.2%, which is 13.2% lower than the average G+C content of the bacterial host (8). All three phages are related to cluster AU phages, with greater than pairwise 82% identity spanning 65% of the genome lengths.

Genomes were annotated using DNAMaster (http://cobamide2.bio.pitt.edu), Glimmer (9), and GeneMark (10), and putative functions were assigned using BLASTP (11), HHPred (12), and Phamerator (13). All genes were transcribed in the forward direction, and no tRNAs were predicted by Aragorn (14). The number of predicted protein-coding genes ranges from 86 to 92, and up to 23% have putative functional assignments. All of the cluster AU phages have a lysis cassette near the left end of the genome. However, Caterpillar, Nightmare, and Teacup lack the putative glycosidase gene present in CapnMurica and Gordon (15) and have an endolysin gene with predicted muramidase activity. No closely linked holin genes were identified. The virion structure and assembly genes include a fused capsid/capsid maturation protease, a conserved feature that is similarly found in cluster AM, BI, DJ, and CC phages, which infect *Arthrobacter*, *Streptomyces*, *Gordonia*, and *Rhodoccoccus*, respectively. Other genes shared among these diverse clusters code for terminase, portal, primase, ATP-dependent helicase, RecB-like exonuclease, and helix-turn-helix (HTH) DNA-binding domain proteins. No integrase or repressor genes were identified, and these phages are predicted to have lytic lifestyles. Interestingly, TMHMM predicted approximately 20 transmembrane proteins coded in each genome (13). These proteins are small (average size, 137 amino acids) and of unknown function. Most have a single transmembrane domain, although some (e.g., Caterpillar gp29) contain as many as five predicted transmembrane domains. Near their right ends, the genomes also have three HNH endonucleases with various sequence similarities.

### Accession number(s).

These phage genomes are available at GenBank with the accession no. MF140401, MF140423, and MF140432.
